# An *in vitro* evaluation of various irrigation techniques for the removal of double antibiotic paste from root canal surfaces

**DOI:** 10.1590/1678-775720160286

**Published:** 2016

**Authors:** Hakan GOKTURK, Ismail OZKOCAK, Fevzi BUYUKGEBİZ, Osman DEMİR

**Affiliations:** 1 - Gaziosmanpasa University, Faculty of Dentistry, Department of Endodontics, Tokat, Turkey.; 2 - Gaziosmanpasa University, Faculty of Medicine, Department of Biostatistics, Tokat, Turkey.

**Keywords:** Endodontics, Lasers, Sodium hypochlorite, Therapeutic irrigation, Ultrasonic therapy

## Abstract

**Objective:**

The aim of this study was to investigate the effectiveness of conventional syringe irrigations, passive ultrasonic irrigation (PUI), Vibringe, CanalBrush, XP-endo Finisher, and laser-activated irrigation (LAI) systems in removing double antibiotic paste (DAP) from root canals.

**Material and Methods:**

One hundred five extracted single-rooted teeth were instrumented. The roots were split longitudinally. Three standard grooves were created and covered with DAP. The roots were distributed into seven groups: Group 1, beveled needle irrigation; Group 2, double side-vented needle irrigation; Group 3, CanalBrush; Group 4, XP-endo Finisher; Group 5, Vibringe; Group 6, PUI; Group 7, LAI. The amount of remaining DAP was scored under a stereomicroscope.

**Results:**

Group 4, Group 6, and Group 7 removed significantly more DAP than the other protocols in the coronal region. Group 7 was more efficient in the middle region; however, no significant difference was found between Group 7 and Group 6. No differences were found between groups in the apical region either, except for the comparisons between groups 7 and 2, and groups 2 and 3.

**Conclusions:**

None of the investigated protocols were able to completely remove the DAP from the grooves. The Vibringe and XP-endo Finisher systems showed results similar to those of conventional needle irrigation.

## INTRODUCTION

The main goal of root canal treatment is to enlarge the root canal system and to eliminate and discharge bacteria from it. For this purpose, numerous instruments, irrigation solutions, and medicaments have been used^12^. Chemomechanical preparation is often selected as the first option to achieve the goal of eliminating the intracanal bacterial population. Although chemomechanical preparation reduces the bacteria population, none of the contemporary techniques can completely clean the root canal system^16^. Therefore, intracanal medicaments are used to eliminate and/or reduce the number of bacteria from the canal^22^.

Calcium hydroxide (CH) is widely used as an intracanal medicament between appointments to enhance the incidence of bacteria free canals. CH has therapeutic properties, is biocompatible, inhibits osteoclastic activity, can dissolve organic tissue, and has regenerative properties^5^ ^,^ ^28^. Despite these advantages, CH is insufficient in completely removing bacteria from root canals^20^.

Antibiotic pastes are another example of intracanal medicament. Triple antibiotic paste (TAP) (composed of ciprofloxacin, metronidazole, and minocycline) or double antibiotic paste (DAP) (contains ciprofloxacin and metronidazole) are commonly used as intracanal medicaments in cases in which CH cannot alleviate the symptoms^9^ ^,^ ^30^.

Previous investigations agree that the medicament should be completely removed from the root canal system before filling. Researchers have shown that remnants of medicaments prevent penetration of sealers or cements into the root canal dentin walls, as they act as a physical barrier along the sealer/dentin interface^1^ ^,^ ^14^. Furthermore, clinical concentrations of CH, DAP, and TAP can lead to a moderate inflammatory reaction in subcutaneous tissues^11^ and are cytotoxic to human dental pulp stem cells^19^. Thus, the complete removal of medicaments from the root canal is an important step in successful root canal treatment.

The most commonly used technique for the removal of medicaments is recapitulation of the root canal with a master apical file at the working length (WL) followed by copious irrigation with ethylenediaminetetraacetic acid (EDTA) and sodium hypochlorite (NaOCl)^24^. Previous studies have reported that the passive ultrasonic irrigation (PUI) and photon-induced photoacoustic streaming (PIPS) techniques removed more medicament than the conventional needle irrigation system^2^ ^,^ ^4^ ^,^ ^8^.

The XP-endo Finisher (FKG, Dentaire SA, La Chaux-de-Fonds, Switzerland) is a newly introduced shape-memory NiTi instrument. This instrument’s design is similar to that of an ISO size #25, 0.00 taper NiTi file. According to the manufacturer, this file improves irrigation solutions by focusing them on the irregular area of the root canal system; this is achieved by providing an expanded reach (6 mm in diameter or 100-fold of an equivalent sized file), and these files do not cut the dentine^10^.

However, it is unknown whether XP-endo Finisher or CanalBrush can remove DAP from the root canal wall. Therefore, the aim of this study was to investigate the efficacy of conventional syringe irrigations, CanalBrush, XP-endo Finisher, Vibringe, PUI, and laser-activated irrigation (LAI) systems in removing DAP from the artificial standardized grooves in the root canal. The null hypotheses were that the removal of DAP was not affected by the [1] section of root canal (third) or the [2] irrigation techniques.

## MATERIAL AND METHODS

This study was reviewed and approved by the Tokat Clinical Research Ethics Committee of the Gaziosmanpaşa University Faculty of Medicine (15 KAEK 225).

**Figure 1:**
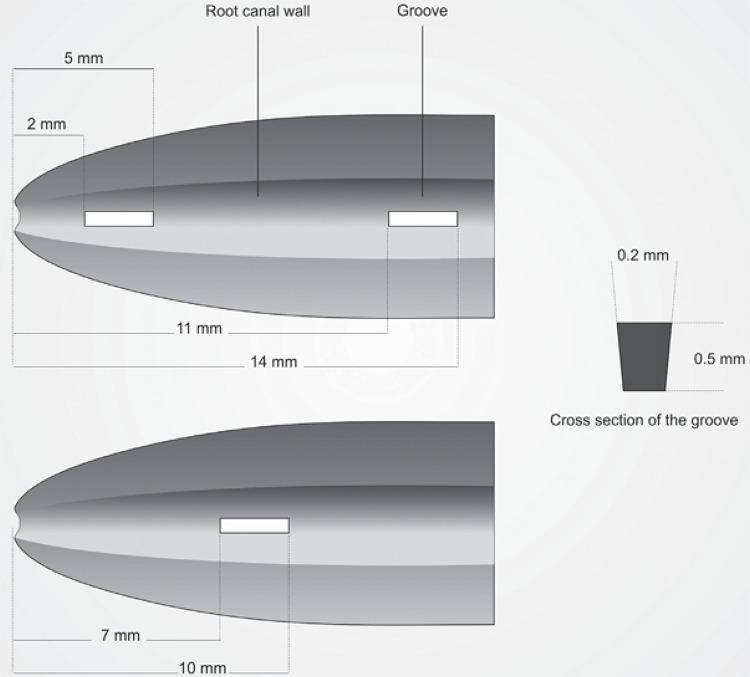
A schematic representation of the location and size of the longitudinal grooves

One hundred and five single-rooted straight human anterior teeth were selected through buccolingual and mesiodistal radiographs, after confirmation that there was one root canal, an intact apex, and no signs of internal/external resorption. The teeth were shortened using a diamond disc under water cooling, and each tooth was given a novel measurement of 15 mm WL using a #10 K-file (Mani Inc., Tochigi, Japan). Canals were prepared with Reciproc rotary files (VDW GmbH, Munich, Germany) and a torque-controlled motor (Silver Reciproc; VDW, Munich, Germany) to a master apical file size of R40 at WL. During preparation, the root canals were irrigated with 10 mL of 2.5% NaOCl (Whitedentmed, Erhan Kimya, İzmir, Turkey), and then they were placed in Eppendorf vials (Labosel, İstanbul, Turkey) filled with silicone impression material (Clinical Zetaplus soft; Zhermack, Badia Polesine, Italy). After the impression material was fully set, the roots were grooved with a diamond disk and split longitudinally without damaging the inner layer of dentine around the canal. A number 1S cavitron tip (Aceton, Merignac, France) was modified to create artificial standardized grooves, which were 3 mm in length, 0.5 mm in depth, and 0.2 mm in width, and were located 2–5 mm from the apex for apical sections, 11-14 mm from the apex for coronal sections, and 7-10 mm from the apex in the opposite part of the middle section (Figure 1). The photographs were taken using a stereomicroscope (Zeiss Stemi 2000-C; Carl Zeiss Microlmaging, Göttingen, Germany) equipped with a digital camera (AxioCam ERc5s, Germany) at 20X magnification. Each of the root halves and grooves were irrigated with 5 mL 17% EDTA (Imicryl Ltd., Konya, Turkey) for 60 sec. and 5 mL 2.5% NaOCl for 60 sec. Then, the teeth were agitated with a tooth brush to remove debris and the smear layer. The root canal was dried with paper points.

Equal amounts of ciprofloxacin (Biofarma, Istanbul, Turkey) and metronidazole (İ.E. Ulagay, Istanbul, Turkey) were mixed with distilled water at a liquid/powder ratio of 1:3 based on the formulations used in the study by Hoshino, et al.^15^ (1996). Each of the grooves was filled with DAP by a modified spreader, and stereomicroscopy was used to confirm that the grooves were fully filled with DAP. Then, while taking care to avoid wax flow into the root canal, the root halves were reassembled with wax, and all the roots were remounted into Eppendorf vials. Next, they were fully filled with DAP using a Lentulo spiral (size 40). The access cavities were temporarily sealed (Cavit, ESPE GMBH, Seefeld, Germany) and stored at 37°C with 100% relative humidity for 2 weeks. After this period, specimens were randomly divided into seven experimental groups (n=15).

For the removal of DAP in all 7 groups, an R40 (VDW, Germany) file at WL and 1 mL 2.5% NaOCl were used to obtain a space for irrigation needles and instruments.

G1 (Beveled Needle): The root canals were irrigated with 10 mL 2.5% NaOCl for 2 min with a 27-gauge beveled dental irrigation needle (Ayset, Adana, Turkey). The tip of the needle was inserted 1 mm short of the WL.

G2 (Double Side-Needle): The irrigation protocol was the same as that in G1 with the exception that a 30-gauge double side-vented needle (i-Tips, i dental, Siauliai, Lithuania) was used (rather than a 27-gauge beveled dental irrigation needle).

G3 (CanalBrush): The root canals were irrigated with 5 mL 2.5% NaOCl and then brushed with a medium size CanalBrush (Coltene/Whaledent GmbHCo. KG, Langenau, Germany) at 600 rpm for 1 min. Then, the root canals underwent a final flush with 5 mL 2.5% NaOCl. The CanalBrush was inserted 1 mm short of the WL and was moved around in small vertical movements.

G4 (XP-endo Finisher): The irrigation protocol was the same as that in G3 with the exception that the XP-endo Finisher (FKG, Switzerland) was used at 800 rpm with 1 Ncm for 1 min (rather than the CanalBrush).

G5 (Vibringe): A 10 mL 2.5% NaOCl solution was delivered and sonically activated via the Vibringe system (Vibringe B. V. Corp, Amsterdam, Netherlands) for 2 min. The needle tip was placed 1 mm short of the WL.

G6 (PUI): Passive ultrasonic irrigation (PUI) was performed using an Irrisafe ultrasonic tip (size 25, 0.00 taper) (Acteon, France), which was driven by an ultrasonic device (Newtron P5; Satelec, Acteongroup, France) 1 mm short of the WL. A 10 mL 2.5% NaOCl solution continuously delivered at a flow rate of approximately 0.16 mL s^-1^ through the unit. It was activated at a power setting of 5 for 1 min.

G7 (LAI): The irrigation protocol was the same as that in G3 with the exception that an Er:YAG laser (Kavo Key 3+, KaVo, Biberach, Germany) with a 2940 nm wavelength was used for 1 min with endodontic tips. The laser parameters were 1 W, 10 Hz, and 100 mJ. When the irrigant in the root canal dropped or vaporized, the canal space was filled with 2.5% NaOCl.

**Figure 2:**
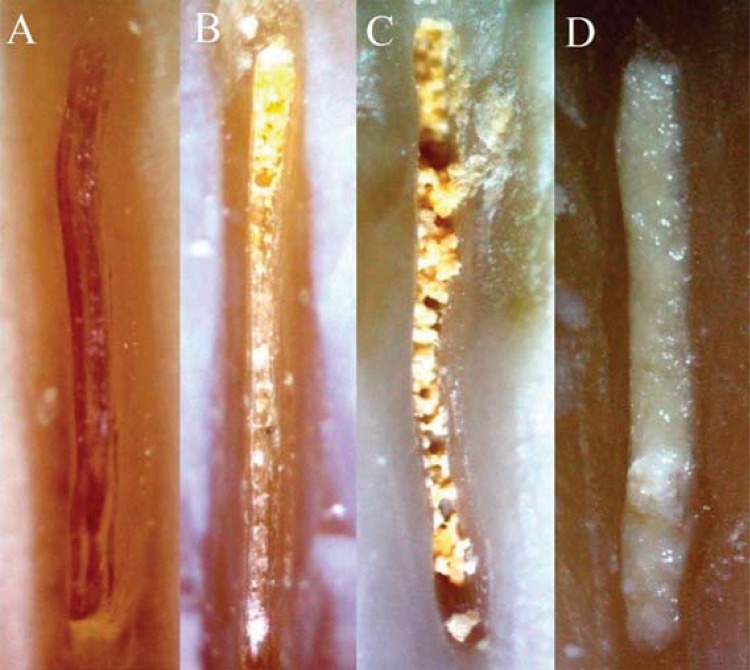
Images of scores: (A) Score 0; (B) Score 1; (C) Score 2; (D) Score 3

The total volume of irrigant for each specimen was set at 11 mL and was delivered at a flow rate of approximately 0.08 mL s^-1^ except for G6 (PUI). The irrigant was delivered into the canal with a 30-gauge double side-vented needle (i dental, Lithuania) except for G1 (beveled needle). The root canals were dried with paper points, and the root halves were separated so that digital photographs could be taken of the canal walls as described above. The digital images obtained at 20X magnification were coded before evaluation to ensure that the evaluators were blinded to their identities. The amount of DAP remaining in the grooves was evaluated by two dentists using a numeric evaluation scale as described by van der Sluis, et al.^32^ (2007). The scoring system was as follows: score 0, the groove is entirely empty; score 1, DAP is present in less than 50% of the groove; score 2, more than 50% of the groove is covered with DAP; and score 3, the groove is completely covered with DAP (Figure 2). Before scoring, the 2 examiners assessed 30 randomly selected specimens together for calibration purposes. In the case of discrepant scores, a consensus was reached by discussion.

Analyses were conducted using commercial software (IBM SPSS Statistics 19, SPSS Inc., IBM Co., Somers, New York, USA). The kappa test was used to determine interexaminer agreement. The Kruskal-Wallis and Mann-Whitney U tests were used to compare the remaining DAP scores. Results were analyzed at 95% confidence intervals, and values of *p* less than 0.05 were considered significant.

## RESULTS

**Figure 3:**
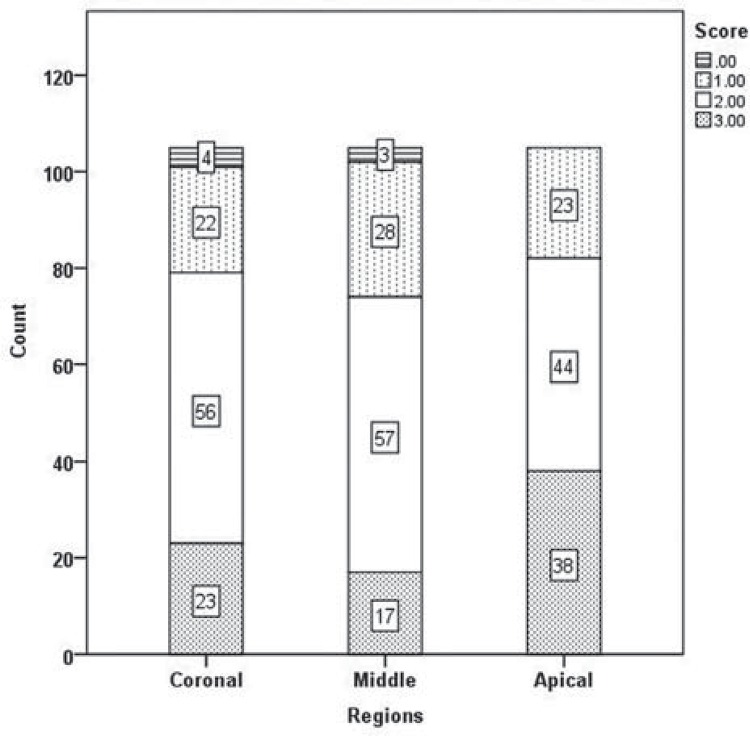
Distribution of scores according to regions

**Figure 4:**
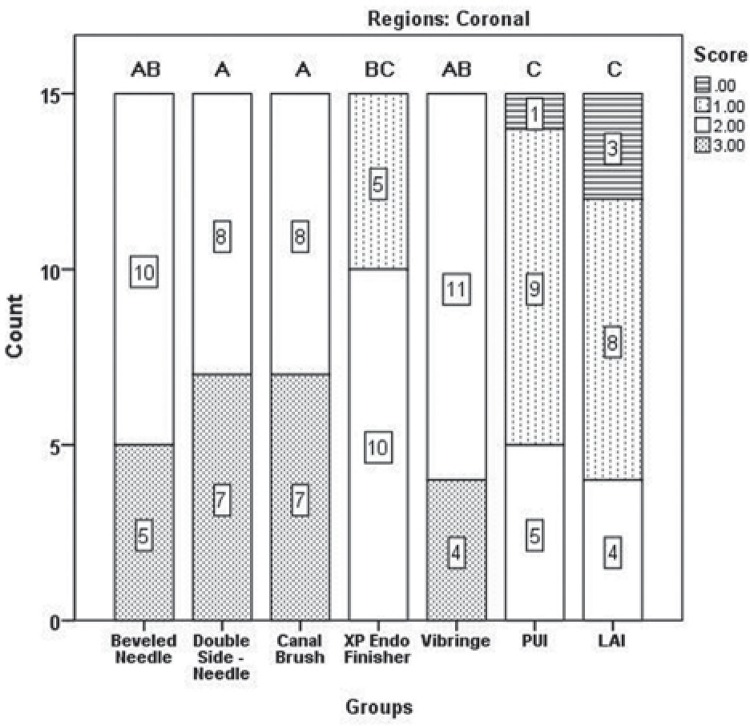
The distribution of scores for double antibiotic paste removal in the coronal third. Different letters denote significant differences between the groups (p<0.05)

**Figure 5:**
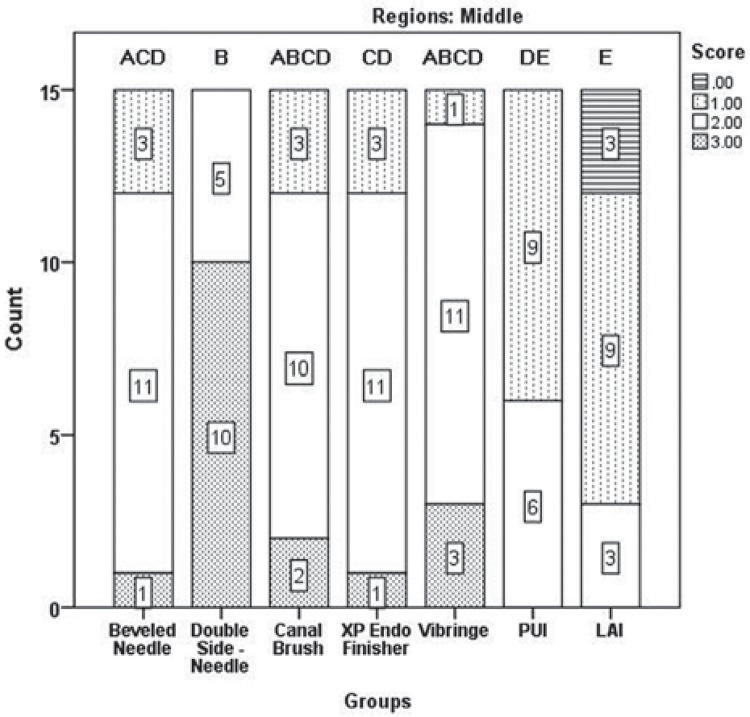
The distribution of scores for double antibiotic paste removal in the middle third. Different letters denote significant differences between the groups (p<0.05)

**Figure 6:**
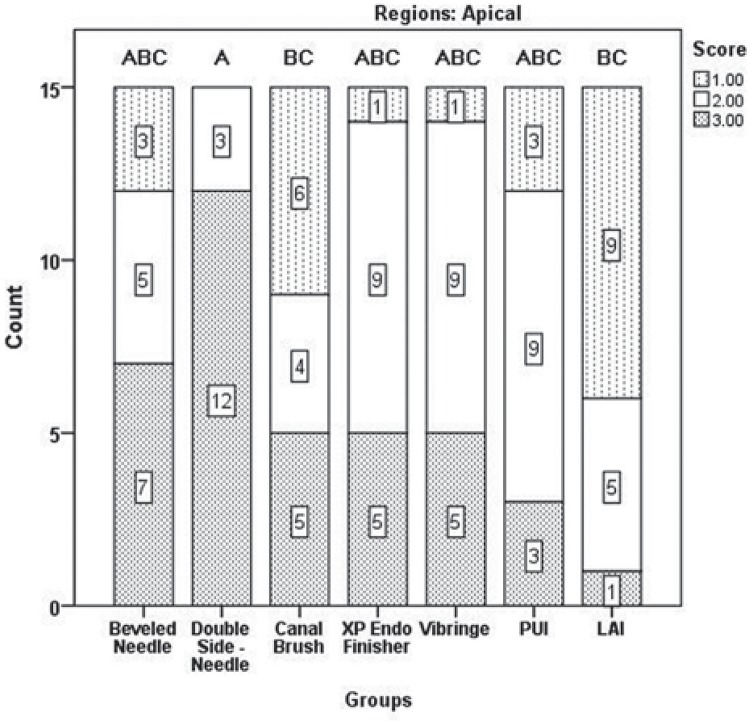
The distribution of scores for double antibiotic paste removal in the apical third. Different letters denote significant differences between the groups (p<0.05)

Results of the two examiners were in good agreement (kappa value=0.907). Figure 3 shows the distribution of scores according to the regions. No irrigation protocols could completely remove all of the DAP remnants. Significant differences were found between tooth regions in terms of paste removal, and more residues were observed in the apical region (Figure 3) (*p<*05). The Kruskal-Wallis test revealed no significant differences between the groups for apical, middle, and coronal thirds (*p*>05), except for the XP-endo Finisher (G4) and PUI (G6) (*p<*05). The XP-endo Finisher (G4), LAI (G7), and PUI (G6) removed significantly more DAP than the other protocols; however, no significant differences were found between these groups in the coronal region (*p*>05) (Figure 4). LAI (G7) was more efficient in the middle region; however, no significant difference was found between the LAI and PUI groups (*p*>05) (Figure 5). It was quite difficult to remove the DAP in the apical region, and no difference was found between groups (*p*>05) except for the comparisons between LAI (G7) and double side needle (G2) and between CanalBrush (G3) and double side needle (G2) (*p*<05) (Figure 6). No significant differences were found between the Vibringe system (G5) and the beveled needle (G1) or double side needle (G2) for all segments (*p*>05).

## DISCUSSION

To our knowledge, no study have compared the effectiveness of needle irrigation, the XP-endo Finisher and CanalBrush in removing DAP from simulated lateral irregularities on root canal walls. Results of this study indicate that no difference was found between tooth regions in terms of paste removal; therefore, the first null hypothesis is accepted. The removal of DAP was not affected by the root canal thirds. However, LAI was superior to double side-needle irrigation in removing DAP at all thirds of the root, and therefore, the second hypothesis is rejected.

Some studies show that CH is insufficient for the elimination of some symptoms, and thus, antibiotic pastes are used as an alternative^9^ ^,^ ^30^ due to their good antimicrobial and biocompatible properties^11^ ^,^ ^15^ ^,^ ^31^. One of the most widely used antibiotic pastes is TAP, which consists of equal portions of metronidazole, ciprofioxacin, and minocycline. However, TAP always causes tooth discoloration^2^. In one study, researchers removed minocycline from the TAP, and developed DAP, which consists of only ciprofloxacin and metronidazole^17^. Although antibiotic pastes have been successfully used in endodontic procedures, they should be completely removed before final root canal obturation to avoid their negative effects, such as tooth discoloration, cytotoxic effects, and the prevention of sealer or cement penetration to root dentin^1^ ^,^ ^2^ ^,^ ^19^. However, it is impossible to completely remove antibiotic pastes from the root canal using conventional irrigation protocol^2^ ^-^ ^4^ ^,^ ^6^ ^,^ ^23^.

There are limited data about the effect of the irrigation protocol on the removal of DAP from the root canal. Akçay, et al.^2^ (2014) investigated the effectiveness of PIPS and an EndoActivator System in removing DAP and TAP from root canal irregularities in the coronal and apical parts of a root canal system. They reported that PIPS was superior for the removal of DAP and TAP, regardless of the groove location, when compared with the EndoActivator and needle irrigation groups. Another study by Arslan, et al.^4^ (2014) investigated the effectiveness of different irrigants with or without ultrasonic activation in removing TAP from simulated root canal irregularities. They reported that PUI with 1% NaOCl was superior to other irrigation techniques in removing TAP. These results are similar to the results obtained in this study.

A previous study by Berkhoff, et al.^6^ (2014) compared positive pressure irrigation, EndoActivator, PUI, and EndoVac from root canal systems by the radiolabeled technique; they concluded that there was no difference in labeled TAP removal among groups. These results are not consistent with those of our study. The differences may reflect different variables in the study design, such as (i) the used medicament, (ii) the irrigation solution’s volume, and/or (iii) measurement technique. In a previous study, it was revealed that minocycline (a compound of TAP) binds to calcium ions via chelation to form an insoluble complex^29^. However, a more recent study reported no significant differences between DAP and TAP removal from artificially created grooves^2^.

In this study, we used a rotary instrument system (XP-endo Finisher) with 2.5% NaOCl as an irrigant for the removal of DAP. Our findings showed that activation of irrigant with a rotary instrument improved the removal of DAP from the artificial grooves; however, this was not statistically different from conventional syringe irrigation. A study conducted by Capar, et al.^8^ (2014) reported that the use of the Self-Adjusting File (SAF) system removed significantly more CH from simulated lateral irregularity in the apical third of root canals than the conventional syringe irrigation. In line with the results of the aforementioned study, Akman, et al.^3^ (2015) have shown that SAF significantly improved the removal of mTAP (metronidazole, ciprofloxacin, and cefaclor) from root canals when compared with conventional syringe irrigation. Unlike rotary or hand instruments, SAF adapts channels in three dimensions, and also allows for copious irrigation during the procedure, which improves its efficiency^3^. Similarly, the superior ability of PUI in removing intracanal medicament from the root canals was attributed to its higher velocity of irrigation solution flow^32^ and its fresh irrigant replacement properties during the procedure^33^.

CanalBrush is a highly flexible endodontic brush made of polypropylene. According to the manufacturer, it helps clean areas of the root canal that cannot be reached by files. To our knowledge, no study has compared the effectiveness of CanalBrush on the removal of antibiotic pastes. However, a recent study reported that no significant difference was found between CanalBrush and PUI on the removal of CH from root canal surfaces^7^. On the other hand, Grischke, et al.^13^ (2014) investigated the efficacy of four different irrigation systems in removing root canal sealer from simulated irregularities on the root canal wall, and reported that PUI is more effective than CanalBrush.

The Vibringe system (Vibringe B. V. Corp, Netherlands) is a device that provides sonic activation of irrigation solutions. This device uses patented sonic flow technology that causes acoustic streaming in the root canal and operates at a lower frequency (2–3 kHz)^25^. Rodig, et al.^25^ (2010) reported that PUI removed significantly more debris from the canal irregularities than did the syringe irrigation and Vibringe System. Johnson, et al.^18^ (2012) reported that Vibringe was not superior to side-vented needle irrigation when comparing debridement efficacies. Similarly, this study showed no significant differences between Vibringe – beveled needle and Vibringe – double side needle for DAP removal for all the segments. However, few studies in the literature examine the effectiveness of Vibringe on the removal of medicaments from the root surface, and therefore, further investigations are needed on this topic.

Sahar-Helft, et al.^27^ (2015) investigated the efficacy of three irrigation protocols (positive-pressure irrigation, PUI, and LAI) on smear-layer removal from human teeth with SEM. An Er:YAG laser at a wavelength of 2940 nm (to 0.5 W, 50 mJ, 10 HZ) was used for 60 sec in the LAI group. They reported that LAI with Er:YAG removed the smear layer from the entire root canal wall better than the other two techniques. Similar findings were observed in a previous study^2^. Although the laser parameters in this study are different from those used in the aforementioned studies, our findings are consistent with the literature. We found that LAI removed more DAP from the root canal surface than the other techniques.

Despite the fact that antibiotic medicaments are commonly used in revascularization and root canal treatments, there are no standardized application times. Er, et al.^9^ (2007) used TAP for intracanal dressing for up to 12 weeks in root canal treatment. In revascularization treatment, the pastes are left in the canal for different periods (1-11 weeks)^21^. Therefore, future studies are needed to thoroughly investigate the application time on the removal capacity.

This study showed that the groove in the apical section gave worse results than the coronal and middle sections, regardless of the irrigation protocol. These results are similar to those obtained by Arslan, et al.^4^ (2014). In contrast, Rodig, et al.^26^ (2010) observed superior results in the apical third, rather than in the coronal third. The conflicts in the published results may be due to the diameter of apical preparation, volume of irrigant, and irrigation protocol used between these studies.

The design of this study has been used by several other investigations^4^ ^,^ ^8^ ^,^ ^13^ ^,^ ^25^ ^,^ ^26^ ^,^ ^32^. However, few studies are investigating the removal of DAP from artificially created grooves^2^. Although the simulated lateral irregularities do not represent the complex structure of a natural root canal system, the groove model has its advantages, which include allowed discrimination between mechanical removal of the medicament and the influence of the irrigant alone, as well as high intraobserver reproducibility^26^. The major limitation of this study is the results obtained from *in vitro* conditions. Thus, future studies should be carried out to confirm the results in clinical conditions.

## CONCLUSION

None of the investigated techniques were able to completely remove the DAP from the artificial standard grooves. However, laser-activated irrigation and PUI may be preferred for the removal of DAP. The Vibringe and XP-endo Finisher systems gave similar results to those of conventional needle irrigation.

## References

[1] Akcay M, Arslan H, Topcuoglu HS, Tuncay O (2014). Effect of calcium hydroxide and double and triple antibiotic pastes on the bond strength of epoxy resin-based sealer to root canal dentin. J Endod.

[2] Akcay M, Arslan H, Yasa B, Kavrik F, Yasa E (2014). Spectrophotometric analysis of crown discoloration induced by various antibiotic pastes used in revascularization. J Endod.

[3] Akman M, Akbulut MB, Aydinbelge HA, Belli S (2015). Comparison of different irrigation activation regimens and conventional irrigation techniques for the removal of modified triple antibiotic paste from root canals. J Endod.

[4] Arslan H, Capar ID, Saygili G, Uysal B, Gok T, Ertas H (2014). Efficacy of various irrigation protocols on the removal of triple antibiotic paste. Int Endod J.

[5] Athanassiadis B, Abbott PV, Walsh LJ (2007). The use of calcium hydroxide, antibiotics and biocides as antimicrobial medicaments in endodontics. Aust Dent J.

[6] Berkhoff JA, Chen PB, Teixeira FB, Diogenes A (2014). Evaluation of triple antibiotic paste removal by different irrigation procedures. J Endod.

[7] Bhuyan AC, Seal M, Pendharkar K (2015). Effectiveness of four different techniques in removing intracanal medicament from the root canals: an in vitro study. Contemp Clin Dent.

[8] Capar ID, Ozcan E, Arslan H, Ertas H, Aydingelge HA (2014). Effect of different final irrigation methods on the removal of calcium hydroxide from an artificial standardized groove in the apical third of root canals. J Endod.

[9] Er K, Kuştarci A, Ozan U, Taşdemir T (2007). Nonsurgical endodontic treatment of dens invaginatus in a mandibular premolar with large periradicular lesion: a case report. J Endod.

[10] FKG swiss endo XP-endo Finisher Documentation.

[11] Gomes-Filho JE, Duarte PC, Oliveira CB, Watanabe S, Lodi CS, Cintra LT (2012). Tissue reaction to a triantibiotic paste used for endodontic tissue self-regeneration of nonvital immature permanent teeth. J Endod.

[12] Gorduysus M, Nagas E, Torun OY, Gorduysus O (2011). A comparison of three rotary systems and hand instrumentation technique for the elimination of Enterococcus faecalis from the root canal. Aust Endod J.

[13] Grischke J, Müller-Heine A, Hülsmann M (2014). The effect of four different irrigation systems in the removal of a root canal sealer. Clin Oral Investig.

[14] Guiotti FA, Kuga MC, Duarte MA, Sant’Anna AJ, Faria G (2014). Effect of calcium hydroxide dressing on push-out bond strength of endodontic sealers to root canal dentin. Braz Oral Res.

[15] Hoshino E, Kurihara-Ando N, Sato I, Uematsu H, Sato M, Kota K (1996). In-vitro antibacterial susceptibility of bacteria taken from infected root dentine to a mixture of ciprofloxacin, metronidazole and minocycline. Int Endod J.

[16] Hülsmann M, Peters OA, Dummer PM (2005). Mechanical preparation of root canals: shaping goals, techniques and means. Endod Topics.

[17] Iwaya SI, Ikawa M, Kubota M (2001). Revascularization of an immature permanent tooth with apical periodontitis and sinus tract. Dent Traumatol.

[18] Johnson M, Sidow SJ, Looney SW, Lindsey K, Niu LN, Tay FR (2012). Canal and isthmus debridement efficacy using a sonic irrigation technique in a closed-canal system. J Endod.

[19] Kamocki K, Nör JE, Bottino MC (2015). Dental pulp stem cell responses to novel antibiotic-containing scaffolds for regenerative endodontics. Int Endod J.

[20] Kvist T, Molander A, Dahlén G, Reit C (2004). Microbiological evaluation of one- and two-visit endodontic treatment of teeth with apical periodontitis: a randomized, clinical trial. J Endod.

[21] Law AS (2013). Considerations for regeneration procedures. Pediatr Dent.

[22] Miller EK, Lee JY, Tawil PZ, Teixeira FB, Vann WF (2012). Emerging therapies for the management of traumatized immature permanent incisors. Pediatr Dent.

[23] Ok E, Altunsoy M, Nur BG, Kalkan A (2015). Effectiveness of different irrigation solutions on triple antibiotic paste removal from simulated immature root canal. Scanning.

[24] Phillips M, McClanahan S, Bowles W (2015). A titration model for evaluating calcium hydroxide removal techniques. J Appl Oral Sci.

[25] Rodig T, Bozkurt M, Konietschke F, Hülsmann M (2010). Comparison of the Vibringe system with syringe and passive ultrasonic irrigation in removing debris from simulated root canal irregularities. J Endod.

[26] Rödig T, Vogel S, Zapf A, Hülsmann M (2010). Efficacy of different irrigants in the removal of calcium hydroxide from root canals. Int Endod J.

[27] Sahar-Helft S, Sarp AS, Stabholtz A, Gutkin V, Redenski I, Steinberg D (2015). Comparison of positive-pressure, passive ultrasonic, and laser-activated irrigations on smear-layer removal from the root canal surface. Photomed Laser Surg.

[28] Siqueira JF, Magalhães KM, Rôças IN (2007). Bacterial reduction in infected root canals treated with 2.5% NaOCl as an irrigant and calcium hydroxide/camphorated paramonochlorophenol paste as an intracanal dressing. J Endod.

[29] Tanase S, Tsuchiya H, Yao J, Ohmoto S, Takagi N, Yoshida S (1998). Reversed-phase ion-pair chromatographic analysis of tetracycline antibiotics. Application to discolored teeth. J Chromatogr B Biomed Sci Appl.

[30] Taneja S, Kumari M (2012). Use of triple antibiotic paste in the treatment of large periradicular lesions. J Investig Clin Dent.

[31] Thibodeau B, Teixeira F, Yamauchi M, Caplan DJ, Trope M (2007). Pulp revascularization of immature dog teeth with apical periodontitis. J Endod.

[32] Van der Sluis L, Wu MK, Wesselink P (2009). Comparison of 2 flushing methods used during passive ultrasonic irrigation of the root canal. Quintessence Int.

[33] Van der Sluis LW, Wu MK, Wesselink PR (2007). The evaluation of removal of calcium hydroxide paste from an artificial standardized groove in the apical root canal using different irrigation methodologies. Int Endod J.

